# The Morphological Characteristics of Mandibular Glands and Tergal Glands in Asian Honeybee (*Apis cerana*) Virgin Queens

**DOI:** 10.3390/insects17080768

**Published:** 2026-07-26

**Authors:** Xinyu Cai, Shuxuan Jing, Yanan Zhu, Jiaxing Huang, Qiaoxia Shang, Guiling Ding

**Affiliations:** 1 State Key Laboratory of Resource Insects, Institute of Apicultural Research, Chinese Academy of Agricultural Sciences, Beijing 100093, China; caixinyu0623@163.com (X.C.); jingshuxuan@caas.cn (S.J.); zhuyanan@caas.cn (Y.Z.); huangjiaxing@caas.cn (J.H.); 2 Key Laboratory of Urban Agriculture in North China, Ministry of Agriculture and Rural Affairs, Beijing University of Agriculture, Beijing 102206, China

**Keywords:** exocrine gland, mandible, mandibular gland area, tergal gland cell

## Abstract

The mandibular glands and the tergal glands have been suggested to play a crucial role in queen pheromone production. However, very few studies have linked changes in mandibular and tergal gland morphology to variations in their pheromone secretions. In this study, we aimed to reveal the age-related morphological development of these glands based on the morphological images and traits. We provided detailed information about how the mandibles, the mandibular glands, and the tergal glands changed in *Apis cerana* virgin queens aged 0 to 10 days. We detected only slight variations in mandible size (length and width of bilateral mandibles) across different age groups. However, the age of virgin queens had a significant effect on the morphological development of their mandibular and tergal glands, as reflected by the mandibular gland area and the morphology of tergal gland cell nuclei. The mandibular gland area of the 7-day-old virgin queens was significantly larger than that of the other age groups. Concurrently, the development of tergal glands reached a stable plateau in queens aged 5 to 7 days. In addition, significant differences were also detected in the surface area of tergal gland cells and the cell nucleus, and the cell nucleus diameter among tergites III, IV, and V. Compared to tergites IV and V, the cell nucleus surface area and the nucleus/cytoplasm index in tergite III exhibited greater variations across different age groups. These results are valuable for expanding our understanding of exocrine gland development in *A. cerana* queens.

## Simple Summary

The queen honey bee keeps order in the colony by producing chemical signals that influence the behavior and reproduction of workers. These signals are produced by several glands located at different body parts. However, little is known about how these glands develop in young virgin queens. In this study, we examined the queens from the day they emerged to 10 days of age and measured the size of their mandibles and the development of the two main signal-producing glands, mandibular glands and tergal glands. We found that mandible size changed very little with age, while the two glands showed clear age-related development. Mandibular glands reached their largest size around seven days after emergence, and the tergal gland reached a stable plateau in queens aged 5 to 7 days. Our results indicate that the first week after emergence is likely to be an important period for gland development in virgin queens. This work improves our understanding of queen biology and may help future studies on queen quality and colony management.

## 1. Introduction

Reproductive division of labor is a defining feature of eusocial insects [1]. A typical honeybee colony is composed of a queen, thousands of workers, and hundreds of drones, which are present seasonally. In most *Apis* species, the queen in the colony specializes in reproduction, and most workers are functionally sterile. Workers can activate their ovaries and start laying eggs that would develop into males in queenless colonies [2]. The queen-produced pheromone is particularly important for maintaining queen dominance and social harmony in the colony [3,4]. The complexity of queen-produced pheromone action stems from its being a multi-glandular, multi-component system. As a multi-glandular bouquet, it is used to mediate many colony activities, such as mate attraction [5], inhibiting the rearing of new queens and the development of ovaries in workers [6,7,8], driving retinue response [9], and stimulating foraging behavior [10].

Queen pheromone is a blend of chemicals secreted by multiple glands, mainly including the mandibular glands, the tergal glands, Dufour’s gland, and Koschevnikov’s gland [11,12,13,14]. The mandibular glands are bilaterally symmetrical glands located in the infra-lateral part of the queen’s head near her mandibles [15]. They extend from the mandibles caudally and are exocrine, large, sack-like glands. Queens’ mandibular glands are lined by variably corrugated simple cuboidal epithelium that surrounds a single large central lumen [16]. Queen pheromone produced by the mandibular gland has been regarded as the main substance involved in maintaining colony homeostasis [17]. Previous studies have identified different main components or amounts of queen mandibular pheromone compounds among honeybee species. In queens of six species (*Apis andreniformis*, *Apis cerana*, *Apis dorsata*, *Apis florea*, *Apis mellifera*, and *Apis nigrocincta*), 4-hydroxy-3-methoxyphenylethanol (HVA) was only detected in mated *A. mellifera* queens [11,18].

Tergal glands, also known as Renner’s glands, have been studied mainly in *A. mellifera* and are primarily found in queens and queenless workers [19,20,21,22,23,24]. Workers exhibit two types (type A and type B) of tergal glands, and type B glands occur in both workers and queens [19]. Type A glands are located along the anterior edge of the tergites II–V and consist of single cells characterized by numerous mitochondria and rough endoplasmic reticulum. Type B glands are bicellular and occur along the posterior edge of tergites II–V [22]. The queens’ tergal glands are mainly located on the underside of tergites III–V in the dorsal part of the abdomen [21]. Significant differences in the diameter of the cell nucleus in tergal glands among tergites III–V were detected in 7-day-old *A. mellifera* queens [25]. The tergal glands of *A. mellifera* queens have been identified as the source of compounds that could evoke retinue behavior from workers [23], attract drones during mating flights [26], and inhibit the development of workers’ ovaries [24].

In the mandibular glands of *A. mellifera* queens, the ultrastructure of the glandular cells evolves with their pheromonal activity during ontogeny. The cytological variations, mainly of the mitochondrial system, were detected during the aging of the queens [27]. The development of the unicellular tergal glands and the morphological changes in tergal gland cells have also been investigated in *A. mellifera* queens of different ages. The surface area of the tergal gland cell nucleus was statistically significantly higher in the 8-day-old queens than in the 1- and 20-day-old queens [21]. One-day-old virgin queens had a significantly larger cell nucleus/cytoplasm ratio of tergal glands than the other age group queens, while 8-, 11-, and 16-day-old queens had similar cell nucleus/cytoplasm index [28]. Besides, previous studies have demonstrated that the compounds secreted by both mandibular and tergal glands are progressively modified during the aging of queens [29,30]. Worker reactions and relative 9-oxo-2-decenoic acid (9-ODA) production in the mandibular gland of *A. m. capensis* virgin queens have been revealed to exhibit a positive queen age-dependent response [30]. De Hazan et al. (1989) attempted to correlate data on ontogenetic changes in the secretions and associated behaviors of workers, indicating that the exocrine secretions from both mandibular and tergal glands of queens contribute to the attraction of workers, and that their intensity is age-dependent [31]. However, very few studies have linked changes in mandibular and tergal gland morphology to variations in their secretions.

Queens become sexually mature and take mating flights 7–10 days after emergence [32]. Detailed research about the morphological characteristics of glands in queens preparing for the mating flight is helpful for better understanding the biology of reproduction of honeybees. Jaremek et al. [21] determined the changes in the morphology of tergal gland cells in uninseminated *A. melliffera* queens. They found that queens aged 8 days exhibited the highest surface area of tergal gland cell nuclei and suggested that the highest metabolic activities of tergal gland cells might be connected with the mating flights they undertake at this age. *A. mellifera* and *A. cerana* are closely related species, having similar characteristics but with some noticeable distinctions [33]. However, we did not find any studies devoted to detailed analyses of the mandibular and tergal glands in *A. cerana* queens.

In this study, we aimed to determine how the morphology of the mandibular and tergal glands changes in differently aged *A. cerana* queens. We hypothesized that the mandibular and tergal glands would show age-dependent development. For the virgin queens aged 0, 3, 5, 7, and 10 days, we measured the length and width of their mandibles and estimated the mandibular gland area, the surface area of tergal gland cells, the surface area and diameter of the cell nucleus, as well as the cell nucleus/cytoplasm index for tergal gland cells. The data were analyzed using linear mixed models (LMMs) and Bayesian binomial mixed-effects models for tergal gland traits that showed significant interaction effects of queen age and tergite position. The results are important for expanding our understanding of exocrine glands and for better understanding the biology of *A. cerana* queens.

## 2. Materials and Methods

### 2.1. Queen Rearing and Sample Collection

The experiment was carried out in the apiary located in Beijing from July to August 2025. Sister queens were reared following the standard queen rearing method [34]. To reduce genetic variation among queens, we grafted one-day-old *A. cerana* larvae from a single mother colony into handmade wax queen cells. These queen cells were kept in three healthy queen-less nursing colonies. These nursing colonies had similar colony strength and were provided with sugar syrup and oilseed pollen ad libitum. Ten days after grafting the larvae, queen cells were transferred from the nursing colonies into an incubator (34.5 °C, 70% RH). Immediately after emergence, each virgin queen attended by 25 nurse workers was put into individual rearing boxes constructed from 420 mL plastic cups following Liang et al. (2025) [35]. The nurse workers of similar age were collected from the brood comb in healthy colonies and were randomly assigned to rearing boxes to minimize potential effects of age and origin of workers. For each box, a 2 mL syringe containing 50% (*w*/*w*) honey–water solution and a 1.5 mL Eppendorf tube with its tip cut containing rapeseed pollen paste were provided. These rearing boxes were kept in an incubator (34 °C, 50% RH). To ensure a sufficient sample size for measuring the changes in morphological traits across different ages, queens were sampled from three independent rearing batches (B1–B3). The virgin queens were collected separately when they were 0, 3, 5, 7, and 10 days old. The queens of each age group were collected from each nursing colony in equal numbers as possible to reduce the colony-level rearing effects. All the queens were stored at −80 °C until subsequent dissection.

### 2.2. Morphological Analyses

#### 2.2.1. Morphological Analyses of the Mandibles and the Mandibular Glands

Following Walsh and Rangel (2018) [15], the bilateral mandibles and the mandibular glands were dissected from each queen’s head under a Leica S9i stereomicroscope (Leica Microsystems Ltd., Wetzlar, Germany). The head was pinned with the frons facing down. Then, the cuticle was dissected, and the paired mandibular glands with the mandibles were gently disentangled from the fat bodies and lifted out. They were put onto a glass slide and photographed. ImageJ v1.54 software (Wayne Rasband, National Institute of Health, Maryland, USA) was used for morphological measurements. The length and width of the mandible were measured following Slater et al. (2020) [36]. The area of the mandibular gland was estimated according to Shawer et al. (2021) [37]. The whole mandibular gland was outlined manually, and subsequently, the total mandibular gland area was detected and measured by ImageJ v1.54. To reduce observer bias, the queens were randomly taken out from the freezer for dissection, and all measurements were performed by the same person. The mandibular glands of differently aged queens are shown in Figure S1.

#### 2.2.2. Morphological Analysis of the Tergal Gland Cells

The abdominal tergites II–VI were dissected from each queen under a Leica S9i stereomicroscope (Leica Microsystems Ltd., Wetzlar, Germany). After the fat bodies were carefully removed, each tergite was placed on a glass slide in 0.6% NaCl and covered with a coverslip. Following Jaremek et al. (2024) [21], the tergal gland tissues were observed under a confocal microscope equipped with BGIMAGING CellView X64 software (FINNER100, FINNER, Xi’an, China). The tergal gland cells were photographed using a camera equipped with a DIC attachment at 200× magnification. For tergites III–V of each queen, we randomly selected 15 tergal gland cells with clearly visible boundaries from each tergite for measurements. For each tergal gland cell, the cell surface area, the cell nucleus surface area, and the diameter of the cell nucleus were measured using ImageJ v1.54 software (Wayne Rasband, National Institute of Health, Maryland, USA). We also calculated the cell nucleus/cytoplasm index (cell nucleus area/cell area) for each cell. The sample sizes of queens and cells for tergal gland traits are shown in Table S1. The tergal gland cells located at tergite III of virgin queens at different ages are shown in Figure S2.

### 2.3. Statistical Analysis

All statistical analyses were conducted in R 4.5.1 (R Core Team, 2025) [38]. Data cleaning and wrangling were conducted using the package “tidyverse” [39]. For the traits of paired mandible (length and width) and mandibular gland area, the effects of queen age (0, 3, 5, 7, and 10 days) and position (left and right) and their interaction effects were analyzed using linear mixed models (LMMs) fitted with the package “lme4” [40]. For all three traits, queen age (0, 3, 5, 7, and 10 days), position (left and right), and their interactions were included as fixed effects, while batch (B1–B3) and queen identity (ID) were included as random intercepts.

For tergal gland traits (cell surface area, cell nucleus surface area, cell nucleus diameter, and cell nucleus/cytoplasm index), data were also analyzed using LMMs fitted with the package “lme4” [40]. The effects of age (0, 3, 5, 7, and 10 days), tergite position (III, IV, and V), and their interactions were included as fixed effects. Batch (B1–B3) and queen ID were included as random intercepts. Taking cell surface area as an example, random intercept variances indicated that a substantial proportion of the total variance was attributable to differences between rearing batches (variance = 109,426; ICC ≈ 0.24), whereas variation among queen individuals was small (variance = 3645; ICC ≈ 0.008). Both batch and queen ID were retained as random effects to account for the hierarchical structure of the data. The same structure of random effects was applied consistently across all mandibular and tergal gland traits analyzed.

Significance of the fixed effect was assessed using Type III F-tests with Satterthwaite’s method via the package “lmerTest” [41]. Post hoc pairwise comparisons were performed using Tukey’s HSD test via the package “emmeans” [42]. For all LMMs, model assumptions of normality and homoscedasticity were verified using diagnostic plots of residuals from the final models. For models with interactions, the model selection process was conducted using single-term deletions, and non-significant interactions were excluded from the model. All figures were produced using the package “ggplot2” [43]. Statistical significance was set at *p* < 0.05.

For tergal gland traits that showed significant interaction effects of queen age and tergite position, we further fitted Bayesian binomial mixed-effects models using the package “brms” [44] to quantify non-linear ontogenetic trajectories. The presence or absence of measurable gland area was modeled using a Bernoulli distribution with a logit link. Fixed effects included linear and quadratic age terms (Age_c + I(Age_c^2)) and their interactions with tergite positions. Although a random intercept for rearing batch was included in the LMMs, it was omitted from the model. Taking the surface area of the cell nucleus of the tergal gland cells, for example, the batch effect comprised only three levels and explained minimal variance [batch-level SD = 3.66, 95% CI (0.12, 13.25)], and its inclusion induced minor divergent transitions in the Hamiltonian Monte Carlo sampling. Removing the batch random intercept eliminated divergences (Rhat < 1.01, Bulk-ESS > 900, Tail-ESS > 1600). The random intercept for queen ID was retained to account for 15 cells measured per queen. Posterior summaries and predictions were extracted and visualized using the package “tidybayes” [45].

By employing Bayesian quadratic mixed-effects modeling, we were able to move beyond simple significance testing and directly estimate the uncertainty surrounding these ontogenetic peaks. This approach allowed us to capture the nuanced, transient nature of tergites’ development, which might have been obscured by traditional frequentist methods.

## 3. Results

### 3.1. The Length and Width of the Mandibles

For the mandible length, there was a significant effect of queen age (LMM, *F* = 3.56, *df* = 4, *p* = 0.009) and position (LMM, *F* = 5.58, *df* = 1, *p* = 0.020) on the mandible length, respectively. For the queen age effect, the mandible length of 0-day-old queens was significantly longer than that of 3-day-old queens (adjusted *p* = 0.019). However, no significant differences were detected among the other age groups (Table 1).

For the mandible width, there was no significant effect of queen age on the mandible width (LMM, *F* = 0.79, *df* = 4, *p* = 0.534). A significant effect of position on the mandible width was detected (LMM, *F* = 9.43, *df* = 1, *p* = 0.003). Comparing the positions, the values of left mandibles were significantly greater than those of right mandibles in both length (*p* = 0.020) and width (*p* = 0.003) (Table 1).

### 3.2. The Mandibular Gland Area of Virgin Queens

There was a significant effect of queen age (LMM, *F* = 55.83, *df* = 4, *p* < 0.001; Table 1), whereas no significant effect of position (LMM, *F* = 1.94, *df* = 1, *p* = 0.167; Table 1) on the mandibular gland area.

**Figure 1:**
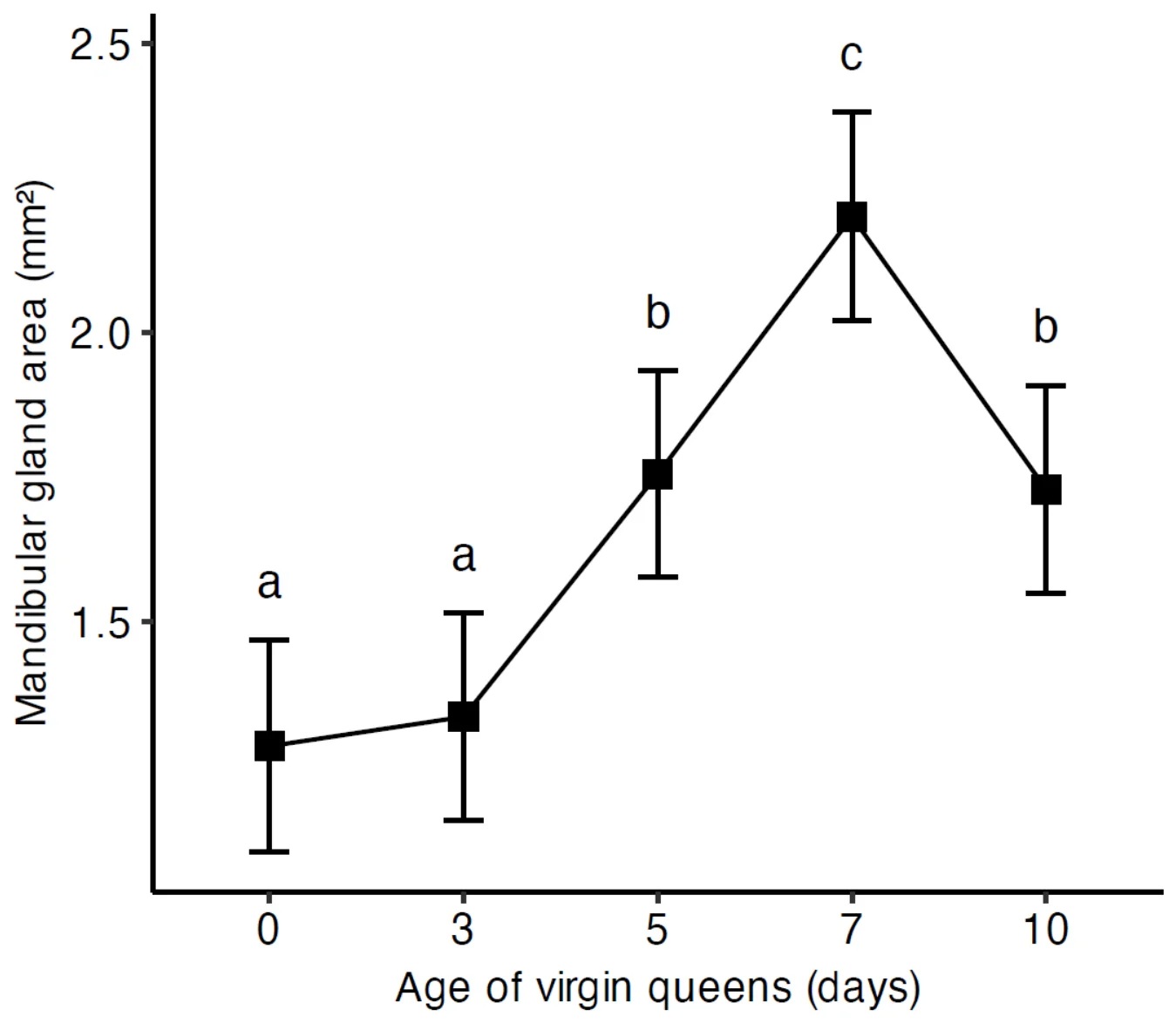
Mandibular gland area of virgin queens at different ages. Error bars indicate the 95% confidence intervals of the estimated marginal means (EMMs). Different letters indicate statistically significant differences in the mandibular gland area between age groups at *p* < 0.05.

The mandibular gland area of the 7-day-old virgin queens was significantly larger than that of the other age groups (all adjusted *p* < 0.001; Figure 1). The mandibular gland areas of 5-day-old and 10-day-old virgin queens were slightly smaller but were significantly larger than those of 0-day-old and 3-day-old queens (all adjusted *p* < 0.001; Figure 1). There were no significant differences in the mandibular gland areas between 0-day-old and 3-day-old queens (adjusted *p* = 0.942) or between 5-day-old and 10-day-old queens (adjusted *p* = 0.995; Figure 1).

### 3.3. The Surface Area of the Tergal Gland Cells in Virgin Queens

Significant differences were detected in the surface area of tergal gland cells among the three tergites (LMM, *F* = 14.30, *df* = 2, *p* < 0.001; Table 2). Regarding the effect of queen age, there was a marginally significant effect (LMM, *F* = 2.30, *df* = 4, *p* = 0.062; Table 2). Among tergite positions, tergite V obtained significantly lower surface area of cells compared to tergite III (adjusted *p* < 0.001) and IV (adjusted *p* = 0.0003) (Table 2).

### 3.4. The Surface Area of the Cell Nucleus of the Tergal Gland Cells in Virgin Queens

A significant interaction effect of queen age and tergite positions was detected in the tergal gland cell nucleus surface area (LMM, *F* = 64.51, *df* = 2, *p* < 0.001; Table S2). Among tergite positions, the nuclear surface area in tergite III was the largest of EMM 433 μm^2^ with 95% CI (388.9, 477) at the age of 7 days (Table S2).

**Figure 2:**
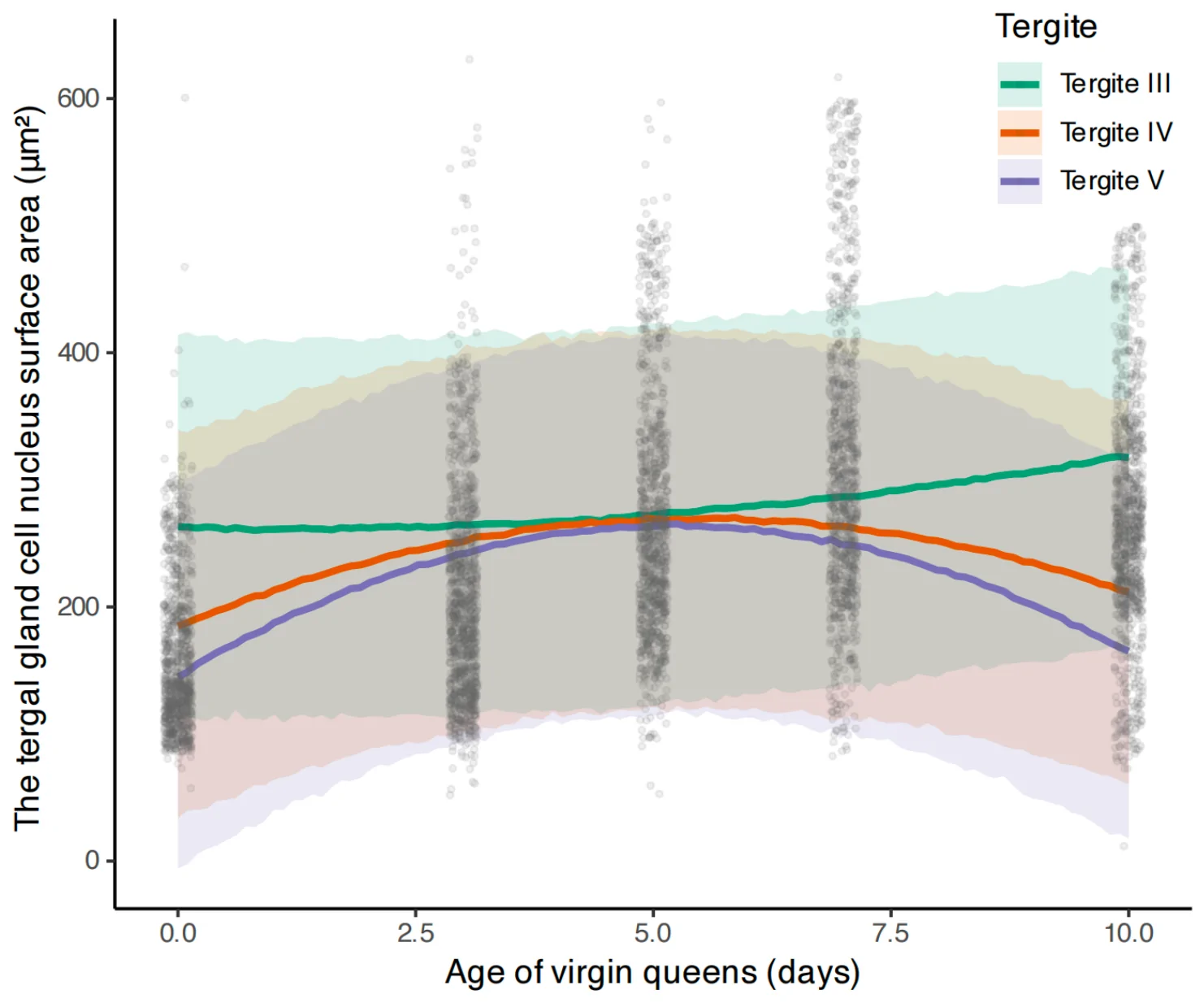
Bayesian posterior predictions of the tergal gland cell nucleus surface area of virgin queens at different ages. Lines represent posterior mean estimates from a Bayesian quadratic mixed-effects model. Shaded areas denote the 95% credible intervals (CrI). Grey jittered points represent raw data. The model reveals a unimodal (hump-shaped) ontogenetic trajectory for all three tergites, peaking around day 5 of adulthood. Tergite III exhibited the largest cell nucleus surface area, followed by tergite IV and tergite V.

Bayesian quadratic mixed-effects modeling estimated that the peak gland area was at approximately 5.5 days for tergite IV (95% CrI: 5.1–5.9) and 5.2 days for tergite V (95% CrI: 5.0–5.5). In contrast, the posterior peak for tergite III occurred around day 5.3 (95% CrI: –22.5 to 16.8), reflecting the model’s limited ability to precisely locate a transient, sharply peaked trajectory that emerges between day 5 and day 7 (Figure 2).

### 3.5. The Diameter of the Cell Nucleus of the Tergal Gland Cells in Virgin Queens

Significant interaction effect of queen age and tergite positions was detected in tergal gland cell nucleus diameter (LMM, *F* = 13.82, *df* = 8, *p* < 0.001; Table S3). Similar to the tergal gland cell nucleus surface area, the tergal gland cell nucleus diameter was the longest in tergite III of EMM 22.52 μm with 95% CI (21.65, 23.4) at the age of 7 days (Table S3).

**Figure 3:**
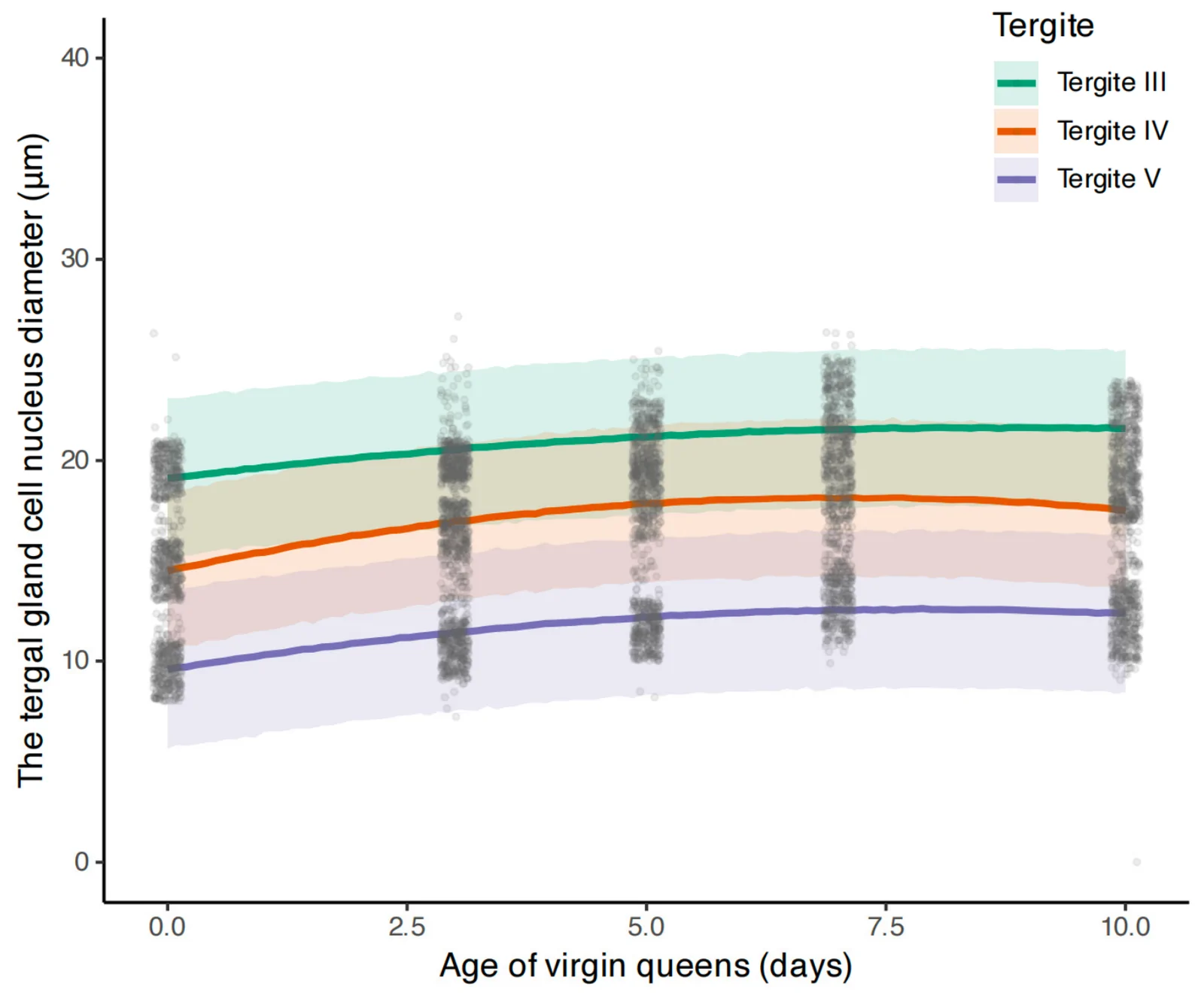
Bayesian posterior predictions of the tergal gland cell nucleus diameter of virgin queens at different ages. Lines represent posterior mean estimates from a Bayesian quadratic mixed-effects model. Shaded bands denote the 95% credible interval (CrI). Grey jittered points represent raw observations. The model reveals a unimodal trajectory for all three tergites, peaking around day 7–9.

Bayesian quadratic mixed-effects modeling confirmed the non-linear, unimodal ontogeny of tergal gland cell nucleus diameter across all three tergites (Figure 3). The posterior probability of a negative quadratic coefficient was >99%, supporting a hump-shaped trajectory for each segment. The estimated peak ages varied significantly among tergites. Tergite III reached its maximum area latest, at approximately 8.9 days post-emergence (95% CrI: 7.4–11.8 days). In contrast, tergite IV peaked earliest at 7.1 days (95% CrI: 6.6–7.7 days), while tergite V showed an intermediate peak at 7.9 days (95% CrI: 7.1–9.2 days) (Figure 3).

### 3.6. The Cell Nucleus/Cytoplasm Index for Tergal Gland Cells in Virgin Queens

Significant interaction effect of queen age and tergite positions was detected in the cell nucleus/cytoplasm index (LMM, *F* = 23.94, *df* = 8, *p* < 0.001; Table S4). Similar to other cell nucleus traits, the value of cell nucleus/cytoplasm index was the highest in tergite III of EMM 0.25 with 95% CI (0.22, 0.28) at the age of 7 days.

**Figure 4:**
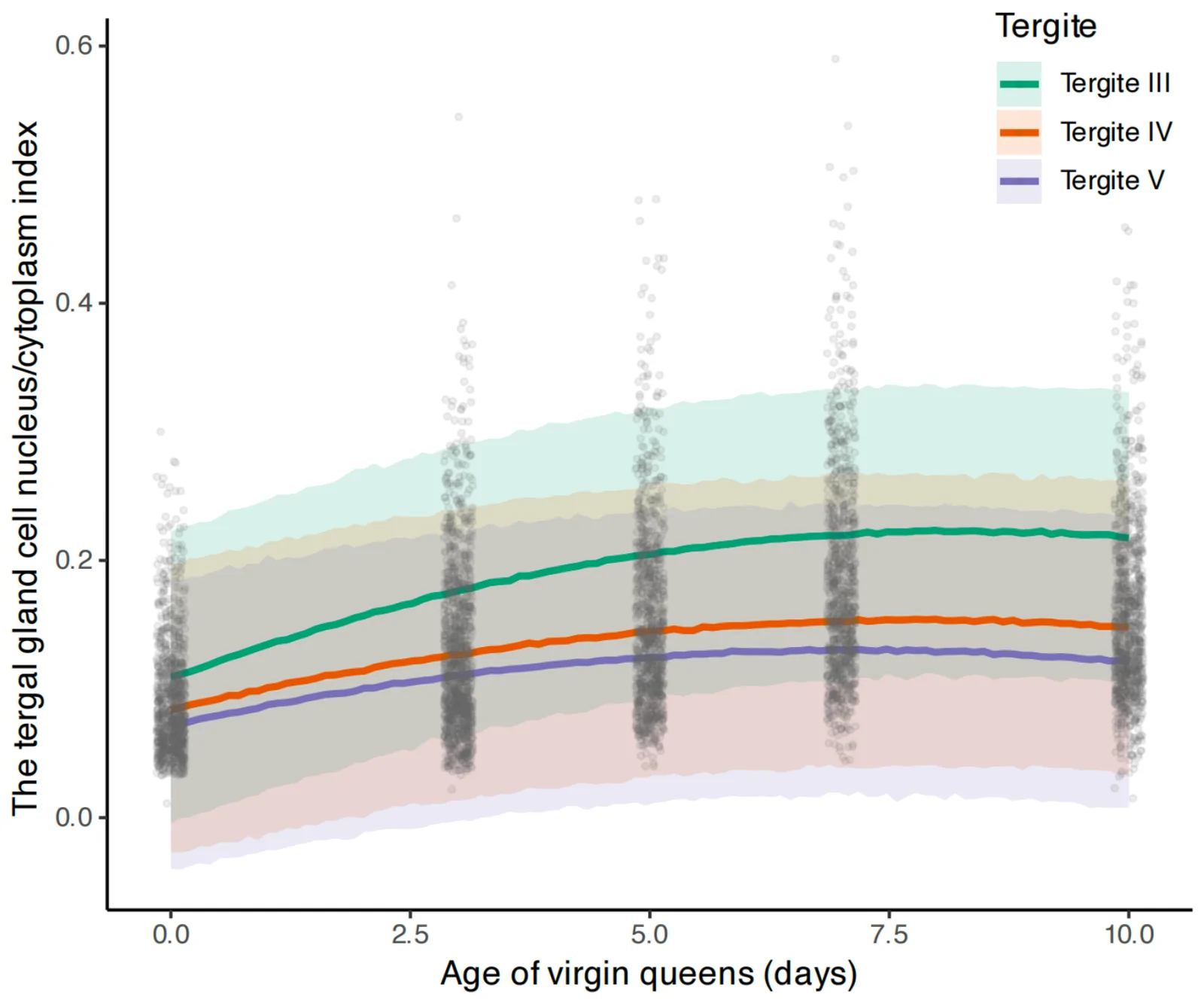
Bayesian posterior predictions of the cell nucleus/cytoplasm index for tergal gland cells of virgin queens at different ages. Lines represent posterior mean estimates from a Bayesian quadratic mixed-effects model. Shaded bands denote the 95% credible interval (CrI). Grey jittered points represent raw observations. The model reveals a unimodal ontogenetic trajectory for all three tergites. Notably, the peak cell nucleus/cytoplasm index occurs sequentially from posterior to anterior: Tergite V peaks earliest (7.2 days), followed by tergite IV (7.9 days), and finally tergite III (8.3 days).

Bayesian quadratic modeling revealed a unimodal trajectory for the cell nucleus/cytoplasm index across all three tergites (Figure 4). Tergite V exhibited the earliest peak in the cell nucleus/cytoplasm index at 7.2 days post-emergence (95% CrI: 6.4–8.4 days). Tergite IV reached its peak subsequently at 7.9 days (95% CrI: 6.9–9.5 days). Tergite III, in contrast, reaching its maximum cell nucleus/cytoplasm index latest at 8.3 days (95% CrI: 7.6–9.3 days) (Figure 4).

## 4. Discussion

The mandibular glands and tergal glands play a crucial role in queen pheromone production, which is vital for the homeostasis of the entire colony. In this study, we provide detailed information on how the mandible, mandibular glands, and tergal glands change as *A. cerana* virgin queens age, which has not been previously addressed. Our study revealed distinct developmental trajectories across different organs: while the size of mandibles (length and width) remained relatively stable, the mandibular and tergal glands underwent significant age-dependent development. The mandibular gland area, as well as the cell nucleus diameter and the cell nucleus/cytoplasm index of the tergal glands, reached their maximum values at around 7 days after emergence. The size of the mandibular gland and the cell nucleus of the tergal gland peaks align with the period when queens naturally become sexually mature for taking mating flight. This indicates that this period may also be a crucial time for the secretion of pheromones. However, we did not conduct chemical analysis to link the morphological change data to pheromone profile variations. Therefore, the functional significance of the morphological changes in the mandibular glands and the tergal glands observed in this study has not been determined. Taking into account that the queen’s performance in the beehive is changing during its lifespan, the morphology of the mandibular glands and the tergal glands needs further study at different stages in mated queens, e.g., just post-mating, brooding queens.

There are reports that queens’ mandible size is affected by genetic differences [46] as well as the queen cell cup size [47]. The right mandible of newly emerged virgin queens of *A. m. carnica* was significantly larger than that of *A. m. jenenitica* queens [46]. Huang et al. (2025) analyzed the effect of queen cell cup diameter on the artificial rearing of *A. cerana* queens and found that the queen’s mandible width was significantly affected by queen cell cup diameter [47]. In our study, virgin queens were reared with the larvae from a single mother colony, ensuring that their genetic backgrounds were identical. In addition, they were reared in wax cups of a similar size. Our results indicated that queens’ age significantly affected mandible length, but had no significant effect on mandible width. Overall, the mandible length and width remained relatively stable among the virgin queens at different ages, which might suggest that the mandible size was determined before adult emergence. Significant differences were detected for mandible length and mandible width between the left and right mandibles. However, position has no significant effect on the mandibular gland area. The biological relevance of this bilateral asymmetry in mandible size remains unclear and should be interpreted cautiously because the absolute changes in mandible size are very limited.

Previous studies indicate that the mandibular gland area is affected by many factors. It was reported that the mandibular gland area of *A. mellifera* workers was significantly correlated with the type of diet and workers’ age [48,49,50]. The mandibular gland area of nurses and foragers of *A. m. carnica* showed significant variations and depended on the rearing season [37]. Another study explored how sun exposure affected the dynamics of *A. mellifera* colonies and queen-rearing success during late winter, revealing that the area of the mandibular glands of workers and queens was significantly influenced by sunlight exposure [51]. Besides, the mandibular gland size of *A. mellifera* queens is associated with pesticide exposure [16], the age of the combs [52], and the protein diets [50]. Our study demonstrated that queen age could significantly affect their mandibular gland area, and virgin queens aged 5 to 10 days old had larger mandibular gland sizes. A larger mandibular gland area may suggest increased potential for pheromone production. In this study, we measured only the total area of the mandibular glands and did not estimate the specific sizes of the mandibular gland epithelium and lumen. We also did not conduct any quantitative analysis of pheromones produced by the mandibular gland. Therefore, further chemical analyses are needed to confirm the relationship between mandibular gland area and the quantity of pheromones produced. A previous study indicated that chemicals participating in the mating process produced by the queen’s mandibular gland were at a higher level in 7-day-old queens [30]. Queens become sexually mature and take mating flights by days 7–10 post-emergence [32]. In our study, the results revealed that the mandibular gland area increased as virgin queens aged after emergence and reached its largest value in 7-day-old queens. Therefore, it seems plausible to hypothesize that there might be a certain correlation between mandibular gland area and mating readiness in queen bees. Future studies assessing mandibular gland development and mating behavior simultaneously can provide us with more precise information about their link.

Wossler et al. (2000) [22] investigated the tergal gland morphology of *A. m. capensis* and *A. m. scutellata* virgin queens, and found that the glands occurred on the posterior edge of tergites II–V. Among the 127 virgin queens that have been analyzed in this study, we only observed a few tergal gland cells (only five) on tergite II for one queen, and none were detected on tergite VI for any of the samples. This was consistent with the results reported in previous studies, stating that tergal glands are mainly located at tergites III–V [28,53]. About the morphological characteristics of the tergal gland cells, our results are consistent with those reported by Strachecka et al. (2022) [25]. The tergal gland cells from different tergites (III, IV, and V) showed similar morphological images but different nuclear diameters. Consistent with the results in *A. mellifera* queens reported by Strachecka et al. (2022) [25], the largest nuclei of tergal gland cells were located on tergite III, and the smallest ones were detected on tergite V. However, further research is still needed to elucidate the relationship between variations in morphological characteristics and the metabolic activities of gland cells.

Our study indicated that the surface area of tergal gland cells did not show the same trend as other morphological characteristics of the tergal gland cells. The surface area of the tergal gland cells remained relatively stable across the three tergites across different age groups of queens. However, the surface area of the cell nucleus, the diameter of the cell nucleus, and the cell nucleus/cytoplasm index showed greater variations. We did not find any studies that analyzed the tergal glands of queens on such a detailed level to compare with our results. Further studies about the ultrastructure of the glands would be helpful to explain the different trends of changes. Jaremek et al. (2024) [21] showed that queens’ age significantly affected the surface area of the tergal gland cell nucleus, which was consistent with our results. They detected that 8-day-old queens had a significantly larger nuclear surface area than 1- and 20-day-old queens. In our study, the maximum nuclear surface area across tergites III-V emerged between day 5 and day 7 post-emergence. We did not encounter a description of changes in the surface area of tergal gland cells in queens in the previous literature.

Azevedo et al. (2007) demonstrated that the Africanized *A. mellifera* queens’ tergal gland development changed according to their age and the emergence location (colonies or ovens) [28]. They detected no significant differences in the tergal glands’ development among tergites III, IV, and V, while the development pattern of the tergal gland was different among queens at different ages. They reported that one-day-old queens had the maximum cell nucleus/cytoplasm index while the six-day-old queens had the minimum cell nucleus/cytoplasm index [28]. In their study, they used the cell nucleus/cytoplasm index as an indicator of the development of tergal glands in queens. Tergal glands with a lower cell nucleus/cytoplasm index were considered more developed because they considered that the nucleus size changed relatively little while the cytoplasm generally increased in size due to the storage of secretion. Contrary to their research findings, our results indicated that the surface area of tergal gland cells remained relatively stable in differently aged queens, while the cell nucleus surface area and cell nucleus/cytoplasm index showed much greater variations. Our study also revealed significant differences in the tergal gland’s morphological traits among the three tergites (III–V). We found that the cell nucleus/cytoplasm index was lowest in 0-day-old virgin queens and highest in 7-day-old queens. Therefore, it remains to be confirmed whether the cell nucleus/cytoplasm index could be used as a universal indicator of tergal gland development.

The secretions of mandibular glands and tergal glands of queenless *A. m. scutellata* workers could be affected by diets [54]. For *A. mellifera* queens, factors including insemination quantity, age, and reproductive status can significantly influence their glands’ chemical profiles [29,54,55,56]. Our study indicated that queens of different ages had different sizes of mandibular and tergal glands. Variations in gland size and morphological traits might be related to the pheromone secretions of the glands. Further investigations are still needed to reveal how age changes the secretion profile of *A. cerana* queens’ glands and to clarify the relationship between glandular development and their secreted compounds. Previous research indicated that queen size was a significant positive predictor of pheromone abundance. They suggested that larger queens might possess larger pheromone source glands [57]. However, without assessing the morphological characteristics of queen size, we could not draw any conclusion regarding the relationship between queen age and queen size.

## 5. Conclusions

This study provides detailed morphological insights into the ontogeny of mandibles, mandibular glands, and tergal glands in *A. cerana* virgin queens during their first 10 days post-emergence. While the mandible size remains relatively stable, both mandibular and tergal glands undergo significant age-dependent development. This study indicates that the size of the tergal gland cell nucleus varies significantly among tergites III, IV, and V. The mandibular gland area is largest in 7-day-old queens. The cell nucleus of tergal gland cells reaches the maximum size at approximately 5–8 days post-emergence. These synchronized developmental peaks align with the period when queens naturally prepare for mating, which might also suggest a critical timeframe for pheromone biosynthesis. Further investigations are needed to reveal how age changes the secretion profile of *A. cerana* queens’ glands and to clarify the relationship between glandular development and their secretions. Ultimately, these findings broaden our understanding of exocrine gland developmental dynamics and the reproductive biology of *A. cerana* queens.

## Figures and Tables

**Table 1:** Estimated marginal means (EMMs) and 95% CI of traits of mandible and mandibular gland for different queen ages and positions.

Factors	Level	Number of Queens (N)	Mandible Length (mm)		Mandible Width (mm)		Mandibular Gland Area (mm^2^)	
			EMM	95% CI	EMM	95% CI	EMM	95% CI
Queen age (days)	0	50	0.878 ^b^	0.855–0.901	0.394	0.364–0.424	1.28 ^a^	1.10–1.47
	3	56	0.859 ^a^	0.835–0.883	0.388	0.357–0.419	1.34 ^a^	1.16–1.51
	5	54	0.862 ^ab^	0.838–0.886	0.386	0.355–0.417	1.76 ^b^	1.58–1.93
	7	44	0.873 ^ab^	0.850–0.895	0.392	0.363–0.421	2.20 ^c^	2.02–2.38
	10	50	0.861 ^ab^	0.838–0.884	0.389	0.360–0.419	1.73 ^b^	1.55–1.91
Position	Left	127	0.871 ^b^	0.847–0.894	0.393 ^b^	0.364–0.423	1.68	1.49–1.87
	Right	127	0.862 ^a^	0.839–0.886	0.386 ^a^	0.357–0.416	1.64	1.45–1.84

Note: EMMs sharing the same letter within each factor of each trait are not significantly different at the 0.05 significance level. Queen age did not show a significant effect on mandible width (LMM, *F* = 0.79, *df* = 4, *p* = 0.534) and position did not show a significant effect on mandibular gland area (LMM, *F* = 1.94, *df* = 1, *p* = 0.167). They were not subjected to post-hoc comparison.

**Table 2:** Estimated marginal means (EMMs) and 95% CI of the surface area of the tergal gland cells (μm^2^) for different queen ages and tergite positions.

Factors	Level	EMM	95% CI
Queen age (days)	0	1714	1219–2208
	3	1749	1255–2242
	5	1774	1280–2268
	7	1804	1310–2298
	10	1783	1289–2277
Tergite position	III	1806 ^b^	1348–2263
	IV	1783 ^b^	1325–2240
	V	1706 ^a^	1248–2163

Note: EMMs sharing the same letter within each factor are not significantly different at the 0.05 significance level; queen age showed a marginally significant effect on mandible width (LMM, *F* = 2.30, *df* = 4, *p* = 0.062) and was not subjected to post-hoc comparison.

## Data Availability

The raw data supporting the conclusions of this article will be made available by the authors on request.

## Supplementary Materials

The following supporting information can be downloaded at https://www.mdpi.com/article/10.3390/insects17080768/s1: Figure S1: The mandibular glands of virgin queens at different ages. Figure S2: The tergal glands located at tergite III of virgin queens at different ages. Table S1: The number of queens and cells measured for tergal gland traits (cell surface area, cell nucleus surface area, cell nucleus diameter, and cell nucleus/cytoplasm index). Table S2: Estimated marginal means (EMMs) and 95% CI of the surface area of the cell nucleus (μm^2^) for different queen ages and tergite positions. Table S3: Estimated marginal means (EMMs) and 95% CI of the diameter of the cell nucleus (μm) for different queen ages and tergite positions. Table S4: Estimated marginal means (EMMs) and 95% CI of the cell nucleus/cytoplasm index for different queen ages and tergite positions.

## Abbreviations

The following abbreviations are used in this manuscript:

QMP	Queen mandibular pheromone
N/C	Nucleus/cytoplasm index
RH	Relative humidity
LMM	Linear mixed-effects model
DIC	Differential interference contrast
PSIS-LOO	Pareto-smoothed importance sampling leave-one-out cross-validation
