# Supplementary material for: The Morphological Characteristics of Mandibular Glands and Tergal Glands in Asian Honeybee (Apis cerana) Virgin Queens
Source: Insects. 2026 Jul 26;17(8):768. doi: 10.3390/insects17080768 (PMC13513001; doi:10.3390/insects17080768)
Supplement: Supplementary file 1 [file insects-17-00768-s001.zip › insects-4332798-supplementary-major back-0701.pdf]

Table S1. The number of queens and cells measured for tergal gland traits (cell surface area, cell nucleus surface area, cell nucleus diameter, and cell nucleus/cytoplasm index).

Factors	Level	Number of queens (N)	Number of cells (N)
Queen age (days)	0	22	990
	3	27	1215
	5	23	1035
	7	22	990
	10	24	1080
Tergite position	III	118	1770
	IV	118	1770
	V	118	1770

Note: For the number of queens, values of queen age represent the count of individual queens at each level. For tergite positions, N indicates the number of queen-tergite observations (each queen was measured for three tergite positions). Total observations = 5310.

Table S2. Estimated marginal means (EMMs) and 95% CI of the surface area of cell nucleus (μm^2) for different queen ages and tergite positions.

Queen age (days)	Tergite position	EMM	95% CI
0	III	196^{c}	151.3-241
	IV	140^{b}	95.8-185
	V	107^{a}	62.6-152
3	III	258^{ef}	214.5-301
	IV	199^{c}	155.8-243
	V	162^{b}	118.1-205
5	III	346^{g}	301.7-390
	IV	233^{de}	189.1-278
	V	199^{c}	154.9-243
7	III	433^{h}	388.9-477
	IV	272^{f}	227.5-316
	V	227^{cd}	182.8-271
10	III	350^{g}	306.1-394
	IV	243^{def}	199-287
	V	198^{c}	154-242

Note: EMMs sharing the same letter are not significantly different at 0.05 significance level.

Table S3. Estimated marginal means (EMMs) and 95% CI of the diameter of cell nucleus (μm) for different queen ages and tergite positions.

Queen age (days)	Tergite position	EMM	95% CI
0	III	19.25^{i}	18.36-20.1
	IV	14.41^{e}	13.52-15.3
	V	9.52^{a}	8.64-10.4
3	III	19.79^{i}	18.94-20.6
	IV	16.47^{f}	15.62-17.3
	V	11.12^{b}	10.27-12.0
5	III	21.03^{j}	20.16-21.9
	IV	17.86^{gh}	16.99-18.7
	V	11.58^{bc}	10.71-12.4
7	III	22.52^{k}	21.65-23.4
	IV	18.23^{h}	17.36-19.1
	V	13.17^{d}	12.3-14.0
10	III	21.13^{j}	20.28-22.0
	IV	17.32^{g}	16.46-18.2
	V	12.04^{c}	11.18-12.9

Note: EMMs sharing the same letter are not significantly different at 0.05 significance level.

Table S4. Estimated marginal means (EMMs) and 95% CI of the cell nucleus/cytoplasm index for different queen ages and tergite positions.

Queen age (days)	Tergite position	EMM	95% CI
0	III	0.126[cdef]	0.100-0.151
	IV	0.094[ab]	0.068-0.119
	V	0.080[a]	0.055-0.106
3	III	0.159[gh]	0.135-0.184
	IV	0.127[def]	0.102-0.151
	V	0.112[bc]	0.087-0.136
5	III	0.207[i]	0.182-0.233
	IV	0.145[fgh]	0.120-0.170
	V	0.127[cde]	0.101-0.152
7	III	0.250[j]	0.225-0.275
	IV	0.165[h]	0.140-0.190
	V	0.138[defg]	0.113-0.164
10	III	0.210[i]	0.185-0.235
	IV	0.147[efgh]	0.122-0.172
	V	0.121[cd]	0.096-0.146

Note: EMMs sharing the same letter are not significantly different at 0.05 significance level.
