# Supplementary material for: The Morphological Characteristics of Mandibular Glands and Tergal Glands in Asian Honeybee (Apis cerana) Virgin Queens
Source: Insects. 2026 Jul 26;17(8):768. doi: 10.3390/insects17080768 (PMC13513001; doi:10.3390/insects17080768)
Supplement: Supplementary file 1 [file insects-17-00768-s001.zip › insects-4332798-supplementary-major back-0720.pdf]

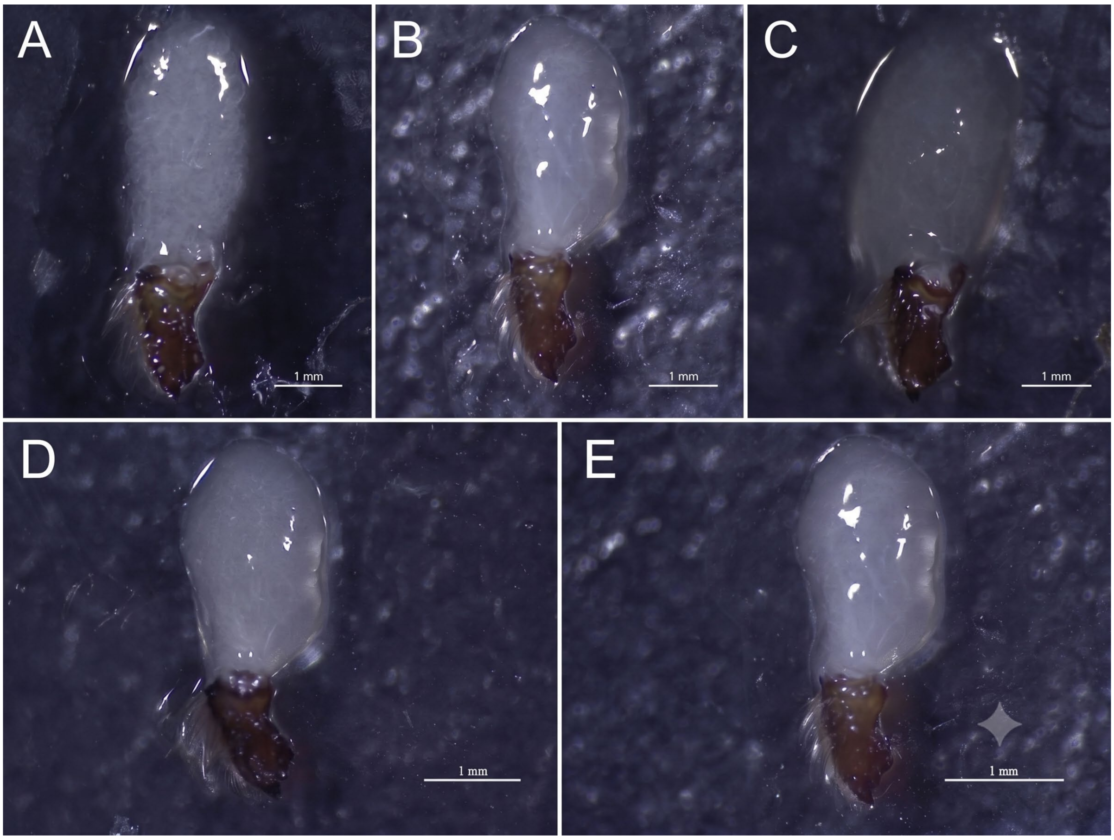

Figure S1 The mandibular glands of virgin queens at different ages. A: 0-day-old, B: 3-day-old, C: 5-day-old, D: 7-day-old, E: 10-day-old.

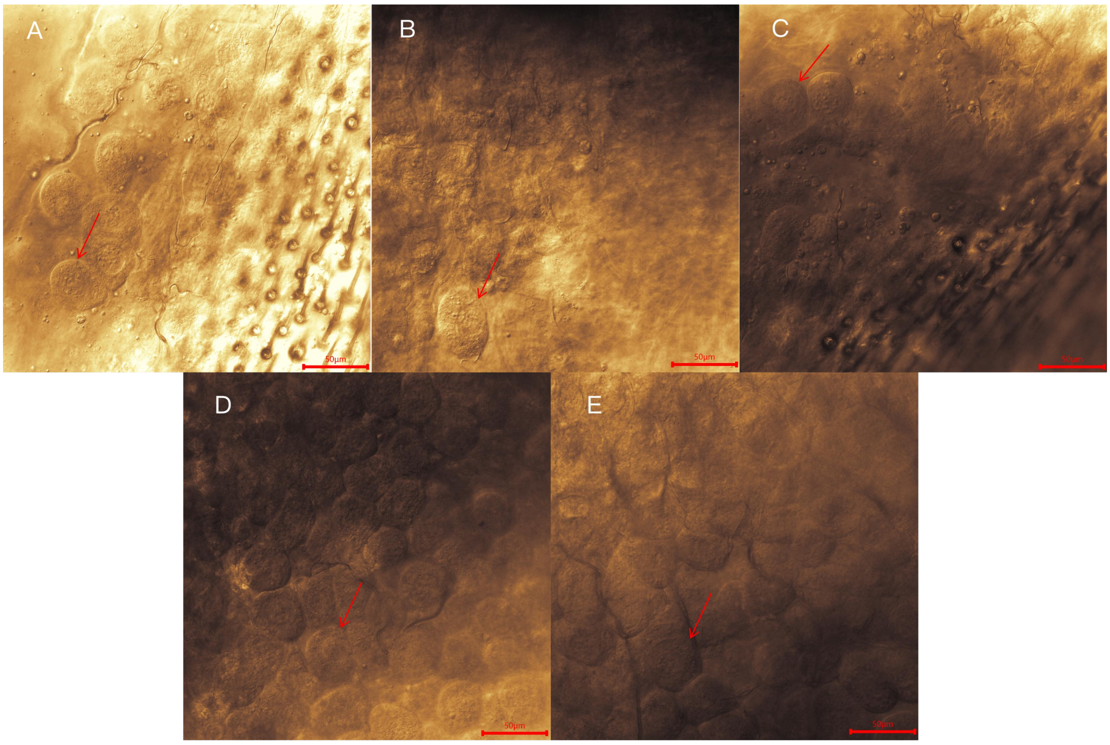

Figure S2 The tergal glands located at tergite III of virgin queens at different ages. A: 0-day-old, B: 3-day-old, C: 5-day-old, D: 7-day-old, E: 10-day-old. The red arrows indicate the tergal gland cells.
